# Motor synergy generalization framework for new targets in multi-planar and multi-directional reaching task

**DOI:** 10.1098/rsos.211721

**Published:** 2022-05-18

**Authors:** Kyo Kutsuzawa, Mitsuhiro Hayashibe

**Affiliations:** Department of Robotics, Graduate School of Engineering, Tohoku University, Sendai 980-8579, Japan

**Keywords:** motor synergy, task generalization, multi-directional reaching, reinforcement learning, optimization

## Abstract

Humans can rapidly adapt to new situations, even though they have redundant degrees of freedom (d.f.). Previous studies in neuroscience revealed that human movements could be accounted for by low-dimensional control signals, known as *motor synergies*. Many studies have suggested that humans use the same repertories of motor synergies among similar tasks. However, it has not yet been confirmed whether the combinations of motor synergy repertories can be re-used for new targets in a systematic way. Here we show that the combination of motor synergies can be generalized to new targets that each repertory cannot handle. We use the multi-directional reaching task as an example. We first trained multiple policies with limited ranges of targets by reinforcement learning and extracted sets of motor synergies. Finally, we optimized the activation patterns of sets of motor synergies and demonstrated that combined motor synergy repertories were able to reach new targets that were not achieved with either original policies or single repertories of motor synergies. We believe this is the first study that has succeeded in motor synergy generalization for new targets in new planes, using a full 7-d.f. arm model, which is a realistic mechanical environment for general reaching tasks.

## 1. Introduction

Humans can generate complex movements (such as object manipulation and locomotion) in diverse situations, using a number of muscles and joints in a coordinated manner. Previous studies have revealed that human movements can be accounted for by a small number of primitive components, known as *motor synergies* [1–3]. The central nervous system is considered to simplify the control problem of complex and redundant musculoskeletal systems by linearly combining a small number of motor synergies [4,5]. Another feature of motor synergies is that the same synergy structures are often observed from various movements performed for different targets [6–9]. Such motor synergies are referred to as *shared synergies*, and they refer to the re-usability of motor synergy structures. It has also been reported that animals, including humans, explore combination patterns of previously acquired motor synergies during motor skill learning [10,11], suggesting that motor synergies can be combined and re-used to deal with new targets. Computationally, seeking motor synergy combinations can reduce the search space in motor learning.

There are previous studies that address the motor synergy structure computationally. The computational approach facilitates the analysis and evaluation of the role of each component using simple models that extract important factors of the actual system. For example, some studies have reported that motor synergy structures can be observed from trajectories acquired by machine learning and optimization: zero-moment point-based locomotion [12], feedback torque integration [13], optimal control [14,15], trajectory optimization [16], reinforcement learning for gait [17] and reaching [18]. These studies provide a methodology for approaching motor synergies without assuming how motor synergies are implemented in animals. However, the aspect of task generalization through the obtained synergy has not been fully discussed. In addition, Rückert & D'Avella proposed a framework that explicitly implemented a shared-synergy structure based on dynamic movement primitives [19,20], movement representations similar to motor synergies [21]. There are also some studies that examined the generalization ability of motor synergies. Al Borno *et al.* evaluated reaching tasks using a musculoskeletal model and reported that a single repertory of motor synergies obtained by trajectory optimization could be generalized to different initial poses and targets [22]. Hagio & Kouzaki demonstrated that a hard-wired motor synergy structure could accelerate motor learning against changes in musculoskeletal structures [23]. These studies reveal the generalization ability of single repertories of motor synergies; however, determining whether combinations of multiple motor synergy repertories can be generalized to new targets remains unclear. In this study, to address this question, we use multiple motor synergy repertories extracted from motor control agents (i.e. policies) that are individually trained with different targets, and we investigate the systematic generalization framework of motor synergies.

This study demonstrates that the combinations of motor synergies extracted from multiple policies can be re-used for generalization of new targets in multi-directional three-dimensional (3D) reaching tasks. We employed several types of target positions in each of which the target positions were located on different planes. The main contribution of this study is to demonstrate the generalization ability of motor synergies extracted from individually learned motor skills. Alternatively, this study provides a framework for explaining and reproducing human’s ability to generalize to new situations by combining previously acquired skills, which will be beneficial to neuroscience, machine learning and robotics.

## 2. Material and methods

### 2.1. Overview

This study demonstrates that the ability of a single repertory of motor synergies can be extended by combining another repertory of motor synergies. In this study, we use *spatio-temporal synergies* to represent the motor synergies as follows:

2.1 $$x(t)\approx \sum_{l=0}^{L-1}h_{l}w_{l}(t),$$

where x(t), $w_{l}(t)$ and *h*_*l*_ indicate the movement, spatio-temporal synergy and activity amplitude, respectively. Considering this equation, it is expected that more diverse movements can be expressed by increasing the number of motor synergies. We demonstrate a more special case in this study. We evaluate how representation ability can be improved by combining motor synergies extracted from different policies. To accomplish that, we construct a method that consists of (i) motor learning to acquire multiple policies, (ii) motor synergy extraction from individual policies, and (iii) generalization to new targets with combined motor synergy repertories.

**Figure 1.**
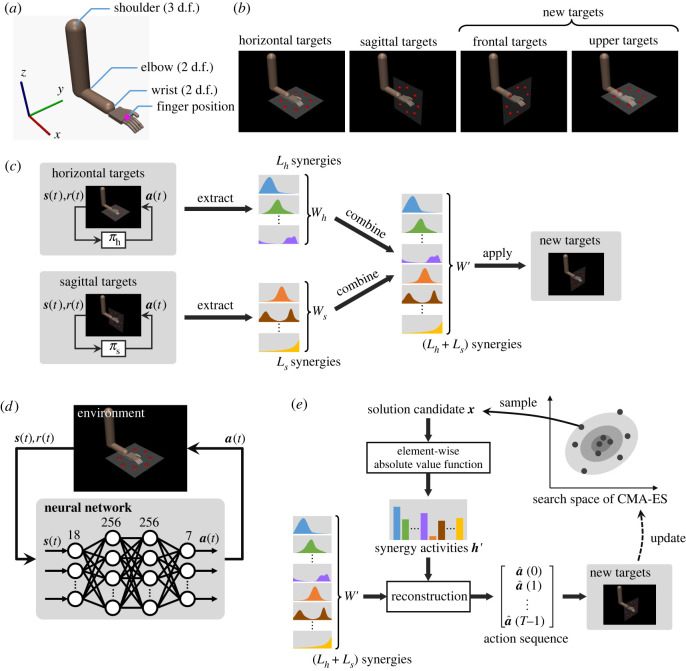
Overview of the method. (*a*) Seven-dimensional arm. (*b*) Reaching targets. The horizontal and sagittal targets are used for reinforcement learning, whereas the frontal and upper targets are regarded as new targets to be generalized. (*c*) The entire process in this study. First, multiple policies, *π*_*h*_ and *π*_*s*_, are learned for the horizontal and sagittal targets, respectively. Thereafter, motor synergy repertories, *W*_*h*_ and *W*_*s*_, are extracted. Finally, the motor synergies are integrated into *W*′, and its activation magnitude is optimized. (*d*) Set-up of reinforcement learning. (*e*) Optimization procedure for generalization. Solution candidates are first sampled from a Gaussian distribution. Thereafter, the solution candidates are converted to synergy activities, which are decoded into an action sequence with combined motor synergies, and the parameters of the Gaussian distribution are updated using the evaluation results.

Figure 1 illustrates an overview of the method. We used tasks as illustrated in figure 1*a*,*b*. The method consists of motor learning, motor synergy extraction, combination, and optimization for new targets (figure 1*c*). First, multiple policies are trained for different types of targets using a deep reinforcement learning method (figure 1*d*). Thereafter, motor synergies are extracted from the time series of the joint torque performed by each policy. Finally, combining the repertories of motor synergies linearly, we applied an optimization method for adjusting motor synergy activities to accomplish other types of targets. These are newly provided to investigate task generalization (figure 1*e*).

### 2.2. Task description

We used a seven-degrees-of-freedom (d.f.) arm such that its kinematics resembled that of human arms. The overview of the arm is illustrated in figure 1*a*. The shoulder, elbow and wrist in this arm have three, two and two rotational joints, respectively. The joints are controlled by torque commands. We used MuJoCo [24] for physics simulation. The joint specifications are described in table 1.

**Table 1.** Joint specifications.

index	lower limit (rad)	upper limit (rad)
1	−1.57	0.70
2	−0.85	1.57
3	−0.85	0.85
4	−1.50	1.05
5	−1.50	1.57
6	−0.50	0.50
7	−1.05	1.05

This study handles a multi-directional reciprocating reaching task; the arm first moves to a target point at a specific time and returns to the initial position at a predetermined finish time. The same type of task is often employed in studies of human arm reaching [3,25]. An observation variable of the task at time *t*, s(t), consists of seven-dimensional joint angles θ(t) (rad), seven-dimensional joint angular velocities $\dot{\theta }(t)\;(\mathrm{rad}\;s^{-1})$, three-dimensional target position $q_{\mathrm{target}}\;(m)$, and normalized time ϕ(t). Here, $q_{\mathrm{target}}$ is fixed for each episode. The normalized time is represented as follows:

2.2 $$\phi (t)=\frac{t}{t_{\mathrm{finish}}},$$

where *t*_finish_ indicates the time when the task should have been terminated. Action a(t) consists of a seven-dimensional joint torque τ(t) (Nm). An immediate reward *r*(*t*) is expressed as follows:

2.3 $$r(t)=\left\{ \begin{array}{cc} \|q_{\mathrm{target}}-q(t){\|}^{2}+k_{1}\|\dot{q}(t){\|}^{2}+k_{2}\|a(t){\|}^{2}\Delta t,\; & t=t_{\mathrm{target}},\;\\ \|q_{\mathrm{initial}}-q(t){\|}^{2}+k_{1}\|\dot{q}(t){\|}^{2}+k_{2}\|a(t){\|}^{2}\Delta t,\; & t=t_{\mathrm{finish}},\;\\ k_{2}\|a(t){\|}^{2}\Delta t,\; & \mathrm{otherwise}.\; \end{array}\right.$$

This reward function forces the arm tip to reach $q_{\mathrm{target}}$ at *t*_target_ and returns to $q_{\mathrm{initial}}$ at *t*_finish_ while attempting to reduce the joint torques during the task. Here, $q_{\mathrm{initial}}={\left[0.24\;m,0.0\;m,0.21\;m\right]}^{⊤}$. $q_{\mathrm{target}}$ indicates the target position to be reached. q(t) and $\dot{q}(t)$ indicate the position and velocity at the centre of gravity of the fingers, respectively. The coefficient values were set to *k*_1_ = 0.002 and *k*_2_ = 0.2. The sampling interval of the policy was set to Δ*t* = 10 ms. Each episode consists of 100 samples of interaction, (i.e. 1 s). In addition, *t*_target_ and *t*_finish_ were set to 50 and 100 (i.e. 0.5 and 1 s), respectively.

We employed four types of target positions: cases in which the target points were on a horizontal, sagittal, frontal and upper-horizontal plane. We refer to them as *horizontal, sagittal, frontal and upper targets*. The target positions in each case are defined as follows:

2.4 $$q_{\mathrm{target}}=q_{\mathrm{initial}}+{\left[-l\text{sin}\theta ,-l\text{cos}\theta ,0\right]}^{⊤},$$

2.5 $$q_{\mathrm{target}}=q_{\mathrm{initial}}+{\left[-l\text{sin}\theta ,0,-l\text{cos}\theta \right]}^{⊤},$$

2.6 $$q_{\mathrm{target}}=q_{\mathrm{initial}}+{\left[0,-l\text{sin}\theta ,-l\text{cos}\theta \right]}^{⊤}$$

2.7 $$\mathrm{and}\;\;\;\;\;\;\;\;q_{\mathrm{target}}=q_{\mathrm{initial}}+{\left[-l\text{sin}\theta ,-l\text{cos}\theta ,z\right]}^{⊤},$$

where

2.8 l=0.15 m,

2.9 $$\theta \in \left\{\frac{2\pi n}{8}|n=0,1,\ldots ,7\right\}$$

2.10 andz=0.05 m.

The target positions are illustrated in figure 1*b*. As explained below, we use only the horizontal and sagittal targets for the initial policy acquisition to acquire each repertory motor synergy, and the other targets are used for synergy-based optimization.

### 2.3. Motor synergy acquisition through reinforcement learning

We first trained policies using proximal policy optimization (PPO) [26], a type of reinforcement learning method. Each policy consists of a neural network with two hidden layers as illustrated in figure 1*d*. Each hidden layer has 256 units with tanh activation. During the training, critic networks with the same hidden layer architecture as the policy networks were used. Another set-up for PPO is detailed in table 2.

**Table 2.** Parameters of reinforcement learning.

item	value
no. training epochs	3000
steps per epoch	10 000
discount factor	0.99
clipping ratio	0.2
*λ* for generalized advantage estimator [27]	0.97
learning rate for the policy network	0.0003
learning rate for the critic network	0.001
number of gradient descent steps per epoch	80

After the training of the policies with PPO, motor synergies are extracted. We used non-negative matrix factorization (NMF) to extract spatiotemporal synergies.

Spatio-temporal synergies indicate primitive patterns of multi-d.f. movements linked both temporally and spatially. It can be extracted with NMF from a matrix of movements in multiple trials, X, as follows:

2.11 X≈WH,

where W and H indicate the motor synergies and their activity amplitudes in individual trials, respectively. Among the various definitions of motor synergies [28], spatio-temporal synergies can compress both spatial and temporal patterns of the time series of actions.

Before extracting motor synergies, it is necessary to pre-process the sequences of *M* d.f. actions that can take both positive and negative values because NMF can only handle non-negative values. First, an action at time *t* in the *n*th trial, $a^{n}(t)$, is converted to two non-negative values, $a_{+}^{n}(t)$ and $a_{-}^{n}(t)$, as follows:

2.12 $$a_{+}^{n}(t)=\max(a^{n}(t),0)$$

and

2.13 $$a_{-}^{n}(t)=-\min(a^{n}(t),0),$$

where $a_{+}^{n}(t)$ and $a_{-}^{n}(t)$ indicate the positive and negative parts of $a^{n}(t)$, respectively. By definition, $a_{+}^{n}(t)-a_{-}^{n}(t)=a^{n}(t)$ holds. Thereafter, an action matrix X is constructed as follows:

2.14 $$X=\left[\begin{array}{ccc} a_{+}^{0}(0) & \ldots & a_{+}^{N-1}(0)\\ \vdots & \ddots & \vdots \\ a_{+}^{0}(T-1) & \ldots & a_{+}^{N-1}(T-1)\\ a_{-}^{0}(0) & \ldots & a_{-}^{N-1}(0)\\ \vdots & \ddots & \vdots \\ a_{-}^{0}(T-1) & \ldots & a_{-}^{N-1}(T-1) \end{array}\right],$$

where *T* and *N* indicate the number of actions in a trial and the number of trials, respectively. Because $a_{+}^{n}(t)$ and $a_{-}^{n}(t)$ can be regarded as *M* × 1 matrices, X has 2*MT* × *N* elements.

Finally, X can be decomposed into spatio-temporal synergies and their activities with NMF as in (2.11). Here, $W\in R^{2MT\times L}$ and $H\in R^{L\times N}$, where *L* is the number of motor synergies. In this study, *T* = 100 and *M* = 7 are used.

### 2.4. Generalization to new targets

By training multiple policies considering the targets, we can obtain multiple repertories of motor synergies. Supposing we have two sets of the motor synergies for the horizontal and sagittal reaching targets, let the numbers of motor synergies be *L*_*h*_ and *L*_*s*_, respectively. Combining them, we can finally use *L*′ ≡ *L*_*h*_ + *L*_*s*_ motor synergies to acquire new movements.

Using these motor synergies, we finally optimized the magnitude of their activities (i.e. *h*_*l*_ in equation (2.1)) for new targets. In summary, the optimization was performed using a numerical optimization method that explores solutions in a way of evolutionary computation.

We optimized the magnitude of the *L*′ motor synergy activities, denoted as $h^{\prime }\in R^{L^{\prime }}$, using the covariance matrix adaptation evolution strategy (CMA-ES) [29,30]. An overview is illustrated in figure 1*e*. The covariance matrix adaptation evolution strategy is a stochastic optimization method that uses a Gaussian distribution to search for a solution. It searches an *L*′-dimensional space in which the coordinates correspond to the activities of motor synergies, and it maximizes the following accumulated reward *G*:

2.15 $$G=\sum_{t=0}^{t_{\mathrm{finish}}}r(t).$$

Considering each optimization step, $\left(4+\lfloor 3\log L^{\prime }\rfloor \right)$ candidates were sampled from a Gaussian distribution, and the parameters of the Gaussian distribution (i.e. the mean vector and covariance matrix) were updated through the evaluation of the sampled candidates. Here, ⌊∙⌋ indicates the floor function. The initial parameters of the Gaussian distribution were set to μ=0 and *σ* = 10^−3^. The CMA-ES explores the entire *L*′-dimensional space containing negative values, whereas the motor synergy activity should be positive. To limit the solutions to positive, a sampled candidate $x\in R^{L^{\prime }}$ was converted to the motor synergy activity $h^{\prime }$ using the element-wise absolute value function, |∙|, as follows:

2.16 $$h^{\prime }=|x|.$$

The motor synergy activity, $h^{\prime }$, was finally decoded to a time series of actions $\hat{a}(0),\ldots ,\hat{a}(T-1)$ as follows:

2.17 $$\left[\begin{array}{c} {\hat{a}}_{+}(0)\\ \vdots \\ {\hat{a}}_{+}(T-1)\\ {\hat{a}}_{-}(0)\\ \vdots \\ {\hat{a}}_{-}(T-1) \end{array}\right]=\left[\begin{array}{cc} W_{h} & W_{s} \end{array}\right]h^{\prime }$$

and

2.18 $$\hat{a}(t)={\hat{a}}_{+}(t)-{\hat{a}}_{-}(t),\;t=0,\ldots ,T-1,$$

where $W_{h}$ and $W_{s}$ indicate the motor synergies in the horizontal and sagittal targets, respectively.

Here we used CMA-ES instead of PPO because of the difference in learning targets. As the motor synergy activities correspond to trajectories one by one, we did not need to optimize sequential decision making but only optimize single variables. Also, we did not use neural networks but directly optimized motor synergy activities. Thus, it was suitable to use a numeric optimization method.

## 3. Results

### 3.1. Motor synergy emergence by deep reinforcement learning

**Figure 2.**
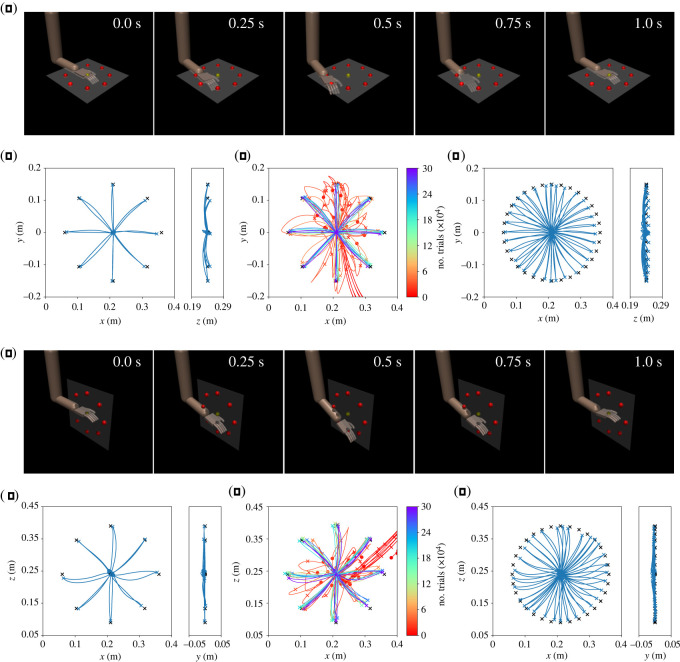
Results in the horizontal and sagittal policies. (*a*–*d*) indicate the horizontal targets, and (*e*–*h*) indicate sagittal targets. (*a*) An example of snapshots in the horizontal targets. (*b*) Trajectories in the horizontal targets. Black cross symbols and bullets indicate *q*_target_ and *q*_initial_, respectively. (*c*) Learning progress. Trajectories at the 0, 10, 20, 30, 50, 100, 150, 200, 250 and 300 (×10^3^)th iterations are shown. (*d*) Trajectories in the various targets with different *θ*, including unlearned ones. (*e*) An example of snapshots in the sagittal targets. (*f*) Trajectories in the sagittal targets. (*g*) Learning progress. Trajectories at the 0, 10, 20, 30, 50, 100, 150, 200, 250 and 300 (×10^3^)th iterations are shown. (*h*) Trajectories in the various targets with different *θ*, including unlearned ones.

Two policies were trained with PPO for the horizontal and sagittal targets (figure 1*b*). We refer to these policies as the *horizontal* and *sagittal policies*, respectively. Figure 2*a*,*e* shows the snapshots of the horizontal and sagittal policies. The movements are also available in electronic supplementary material, movie S1. Also, figure 2*b*,*f* shows the trajectories performed by the horizontal and sagittal policies. In addition, the trajectories during the training are shown in figure 2*c*,*g*. The errors at *t* = *t*_target_, *t*_finish_ in the eight learned targets are summarized in table 3.

**Table 3.** Reaching errors. Mean values and standard deviations are described. Italics type indicates that the mean error in $q_{\mathrm{target}}$ is less than 1 cm.

target type	method	$q_{\mathrm{target}}$ (cm)	$q_{\mathrm{initial}}$ (cm)
horizontal targets	horizontal policy	*0.781 ± 0.494*	*0.419 ± 0.110*
sagittal targets	sagittal policy	*0.863 ± 0.310*	*0.890 ± 0.296*
frontal targets	optimization results (*L*_*h*_ = 5, *L*_*s*_ = 4)	*0.621 ± 0.331*	*0.411 ± 0.164*
	optimization results (*L*_*h*_ = 5, *L*_*s*_ = 0)	6.49 ± 4.12	2.44 ± 2.06
	optimization results (*L*_*h*_ = 0, *L*_*s*_ = 8)	9.21 ± 5.07	0.492 ± 0.152
	horizontal policy	9.52 ± 5.69	0.618 ± 0.230
	sagittal policy	9.46 ± 5.51	0.667 ± 0.210
upper targets	optimization results (*L*_*h*_ = 5, *L*_*s*_ = 5)	*0.754 ± 0.342*	*0.957 ± 0.438*
	optimization results (*L*_*h*_ = 8, *L*_*s*_ = 0)	3.81 ± 0.577	1.69 ± 0.165
	optimization results (*L*_*h*_ = 0, *L*_*s*_ = 6)	9.50 ± 4.68	0.943 ± 0.380
	horizontal policy	4.97 ± 0.288	0.483 ± 0.177
	sagittal policy	9.55 ± 5.11	0.861 ± 0.331

To further confirm the ability of the policies, we evaluate other target positions using different target angles *θ* as follows:

3.1 $$\theta \in \left\{\frac{2\pi n}{32}|n=0,1,\ldots ,31\right\},$$

in which 24 targets have not been given during training. Figure 2*d*,*h* shows that 32 trajectories performed horizontal and sagittal policies.

**Figure 3.**
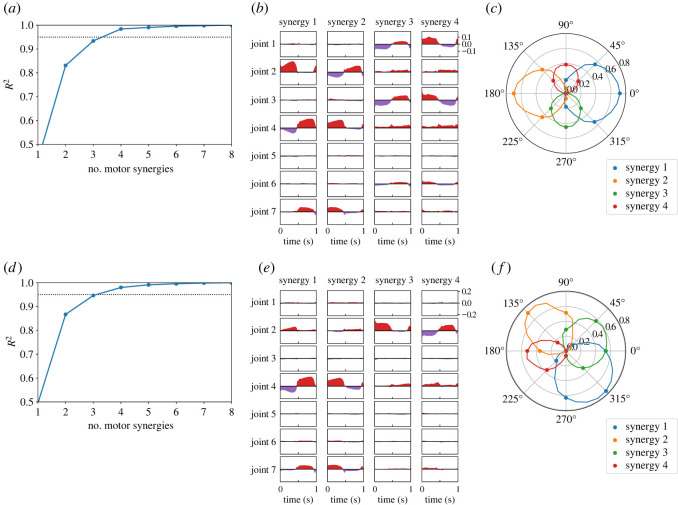
Motor synergies extracted from the horizontal and sagittal policies. (*a*–*c*) Correspond to the results in the horizontal targets, whereas (*d*–*f*) correspond to results in the sagittal targets. (*a*,*d*) Reconstruction performance of motor synergies. (*b*,*e*) Waveforms of motor synergies. The red and purple waves correspond to the positive and negative components of the joint torque, respectively. (*c*) Synergy activities for the target directions *θ* in equation (2.4). $270^{\circ }$ and $180^{\circ }$ correspond to the *x*- and *y*-axes, respectively. In addition, the plots and curves correspond to the motor synergy activities of the 8- and 32-directional reaching movements, respectively. (*c*) Synergy activities for the target directions *θ* in equation (2.5). $270^{\circ }$ and $180^{\circ }$ correspond to the *x*- and *z*-axes, respectively.

We extracted synergy structures from the actions (i.e. joint torques) performed by the horizontal and sagittal policies. Considering the eight trials for each policy, we evaluated the *R*^2^-values to quantify the reconstruction performance for various motor synergies. The *R*^2^-values are computed as follows:

3.2 $$R^{2}=1-\frac{\sum_{t=0}^{T-1}\|a(t)-\hat{a}(t){\|}^{2}}{\sum_{t=0}^{T-1}\|a(t)-\bar{a}{\|}^{2}},$$

where a(t) and $\hat{a}(t)$ indicate the original and reconstructed actions at time *t*, respectively, and $\bar{a}$ indicates the mean values of the actions over the trial. Figure 3*a*,*d* shows *R*^2^-values of the motor synergies extracted from the horizontal and sagittal policies. Four or more motor synergies achieved *R*^2^ > 0.95 in both the horizontal and sagittal policies.

Figure 3*b*,*e* shows the waveforms of the four motor synergies extracted from eight reaching movements shown in figure 2*b*,*f*. Regarding the figures, the red positive values correspond to the components of ${\hat{a}}_{+}(t)$, whereas the purple-coloured negative values indicate the components of ${\hat{a}}_{-}(t)$. Moreover, figure 3*c*,*f* shows the synergy activities for the target position’s directions *θ*. Considering the figures, the marked points correspond to the learned targets shown in figure 2*b*,*f*, whereas the curved lines correspond to the 32 targets shown in figure 2*d*,*h*.

### 3.2. Generalization to new target positions with motor synergies

**Figure 4.**
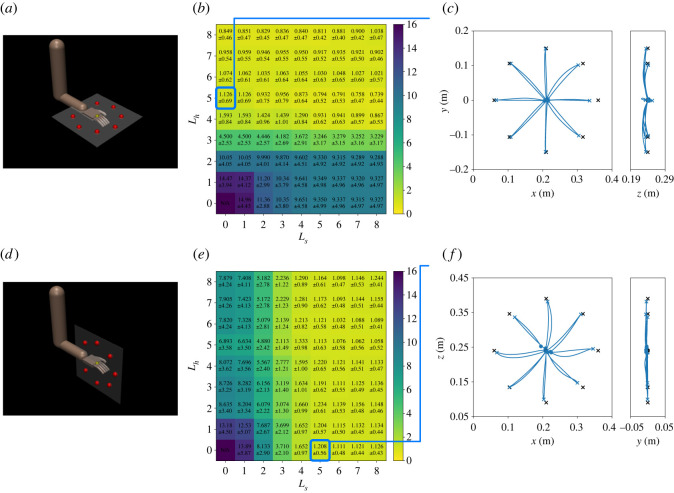
Optimization results in learned targets; (*a*–*c*) correspond to the results in the horizontal targets, whereas (*d*–*f*) correspond to results in the sagittal targets. (*a*) Horizontal targets. (*b*) Error map in $q_{\mathrm{target}}$ (cm). (*c*) Trajectories when *L*_*h*_ = 5 and *L*_*s*_ = 0. (*d*) Sagittal targets. (*e*) Error map in $q_{\mathrm{target}}$ (cm). (*f*) Trajectories when *L*_*h*_ = 0 and *L*_*s*_ = 5.

We optimize the magnitude of the motor synergy activities $h^{\prime }$ using the method illustrated in figure 1*e* to obtain new movements. First, we applied the method of the reaching tasks to the horizontal and sagittal targets to investigate the ability of the motor synergies. Figure 4 shows the results in the horizontal and sagittal targets, which were used in reinforcement learning. Figure 4*b*,*e* shows the errors in $q_{\mathrm{target}}$ with varying numbers of motor synergies. The trajectories in the horizontal targets in *L*_*h*_ = 5 and *L*_*s*_ = 0 are shown in figure 4*c*. In addition, the trajectories in the sagittal targets in *L*_*h*_ = 0 and *L*_*s*_ = 5 are shown in figure 4*f*.

**Figure 5.**
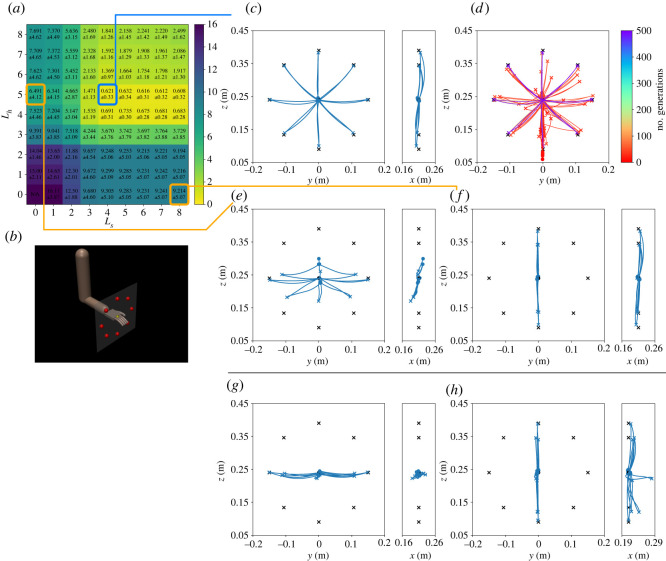
Optimization results in the frontal targets (new targets). (*a*) Error map in $q_{\mathrm{target}}$ (cm). (*b*) Frontal targets. (*c*) Trajectories when *L*_*h*_ = 5 and *L*_*s*_ = 4. (*d*) Learning progress. Trajectories at the 0, 10, 20, 30, 40, 50, 100, 150, 200, 300, 400 and 500th generations are shown. (*e*) Trajectories when *L*_*h*_ = 5 and *L*_*s*_ = 0. (*f*) Trajectories when *L*_*h*_ = 0 and *L*_*s*_ = 8. (*g*) Trajectories by the horizontal policy. (*h*) Trajectories by the sagittal policy.

Subsequently, we evaluated the method in the frontal targets, which can be regarded as new targets in a new plane. Figure 5 shows the results. Figure 5*a* shows the reaching errors in $q_{\mathrm{target}}$, changing the number of motor synergies, *L*_*h*_ and *L*_*s*_. Considering the results, the case with *L*_*h*_ = 5 and *L*_*s*_ = 8 resulted in the smallest error in $q_{\mathrm{target}}$. Figure 5*c* shows the trajectories in *L*_*h*_ = 5 and *L*_*s*_ = 4, where the average error is 0.621 cm; This was not the smallest error, whereas the difference from the smallest error was 0.013 cm, which was approximately 4% of the standard deviation. The movements in *L*_*h*_ = 5 and *L*_*s*_ = 4 are available in electronic supplementary material, movie S1. Figure 5*d* shows the trajectories during the optimization. Furthermore, considering the results when only single motor synergy repertories are used, the cases with *L*_*h*_ = 5 and *L*_*s*_ = 8 resulted in the smallest errors. The trajectories are shown in figure 5*e*,*f*. Comparatively, the trajectories obtained by the policies are shown in figure 5*g*,*h*. The errors at *t* = *t*_target_, *t*_finish_ are summarized in table 3.

**Figure 6.**
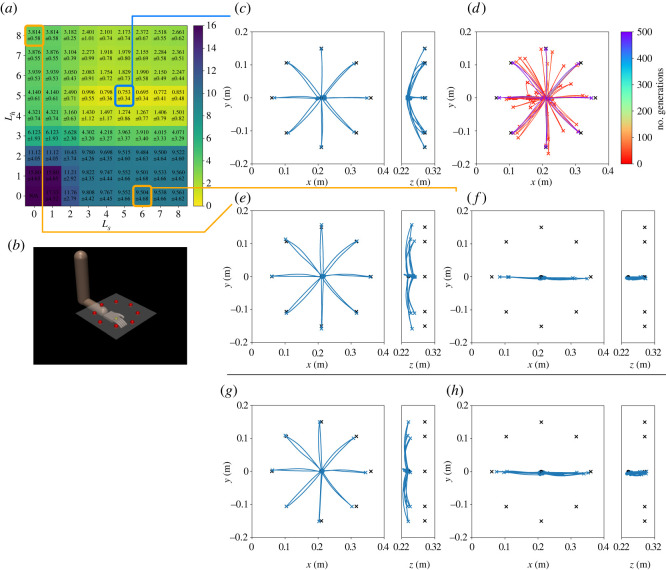
Optimization results in the upper targets (new targets). (*a*) Error map in $q_{\mathrm{target}}$ (cm). (*b*) Frontal targets. (*c*) Trajectories when *L*_*h*_ = 5 and *L*_*s*_ = 5. (*d*) Learning progress. Trajectories at the 0, 10, 20, 30, 40, 50, 100, 150, 200, 300, 400 and 500th generations are shown. (*e*) Trajectories when *L*_*h*_ = 8 and *L*_*s*_ = 0. (*f*) Trajectories when *L*_*h*_ = 0 and *L*_*s*_ = 6. (*g*) Trajectories by the horizontal policy. (*h*) Trajectories by the sagittal policy.

Moreover, the same evaluation is conducted for the upper targets, which can be regarded as new targets in a new plane by sliding the horizontal plane. Figure 6*a* shows the reaching errors in $q_{\mathrm{target}}$, changing the number of motor synergies, *L*_*h*_ and *L*_*s*_. Figure 6*c* shows the trajectories in *L*_*h*_ = 5 and *L*_*s*_ = 5, where the average error is 0.753 cm, and figure 6*d* shows the trajectories during the optimization. The movements in *L*_*h*_ = 5 and *L*_*s*_ = 5 are also available in electronic supplementary material, movie S1. Furthermore, considering the results of when only single motor synergy repertories are used, the cases with *L*_*h*_ = 8 and *L*_*s*_ = 6 resulted in the smallest errors. The trajectories are shown in figure 6*e*,*f*. Comparatively, the trajectories obtained by the policies are shown in figure 6*g*,*h*. The errors at *t* = *t*_target_, *t*_finish_ are summarized in table 3.

### 3.3. Transferability to different arms

We conducted an additional simulation to evaluate if the results so far are consistent when arm models are even different. In human studies, measured data usually contain movements of multiple subjects who have different kinematic parameters such as link lengths. Here, three arm models with different link lengths were used: the original model, a shorter-arm model and a longer-arm model. The link parameters are listed in table 4. Firstly, policies were trained for the shorter- and longer-arm models by using PPO with the same hyper-parameters as in table 2. In policy learning, target positions were calculated with equations (2.4) and (2.5), whereas $q_{\mathrm{initial}}$ varied depending on the arm lengths. We then extracted motor synergies from the trajectories of each arm model and optimized their activities with CMA-ES for the original arm model.

**Table 4.** Link lengths of arm models.

model name	shoulder-to-elbow (m)	elbow-to-wrist (m)
original model	0.36	0.27
shorter-arm model	0.31	0.22
longer-arm model	0.41	0.32

**Figure 7.**
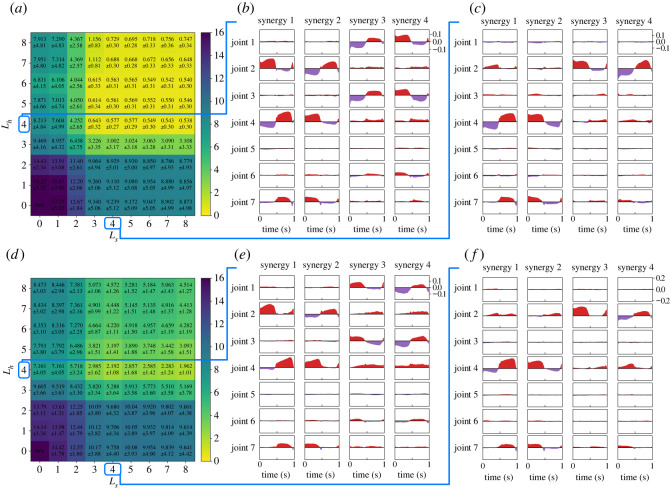
Optimization results in the frontal targets when different arm models were used in synergy extraction. Note that the original arm model was used in optimization. (*a*) Error map in $q_{\mathrm{target}}$ (cm) when motor synergies of the shorter-arm model were applied to the original arm model. (*b*) Motor synergies when the shorter-arm model is reaching the horizontal targets. (*c*) Motor synergies when the shorter-arm model is reaching the sagittal targets. (*d*) Error map in $q_{\mathrm{target}}$ [cm] when motor synergies of the longer-arm model were applied to the original arm model. (*e*) Motor synergies when the longer-arm model is reaching the horizontal targets. (*f*) Motor synergies when the longer-arm model is reaching the sagittal targets.

Figure 7 shows the results in the frontal targets. Compared with figure 5*a*, the motor synergies by the shorter-arm model brought about slightly smaller errors, whereas the motor synergies by the longer-arm model brought about larger errors. When the motor synergies from the shorter-arm model were used, the error was 0.577 ± 0.27 cm in *L*_*h*_ = *L*_*s*_ = 4. In contrast, when the motor synergies from the longer-arm model were used, the error was 2.192 ± 1.08 cm in *L*_*h*_ = *L*_*s*_ = 4; this was approximately three times larger than that observed in figure 5*a*.

## 4. Discussion

### 4.1. Performance of the learned movements

The horizontal and sagittal policies trained with reinforcement learning demonstrated high performance and generalization ability; nonetheless, there were limitations. It is be observed that these policies have learned the reaching task and have demonstrated the generalization ability for the intermediate target positions between the trained ones (figure 2 and table 3). By contrast, when these policies are applied without a combination of policies, the performance is greatly degraded in the frontal and upper targets as shown in figure 5*g*,*h*, 6*g*,*h* and table 3. The difference in the performance arises from whether the new targets are the interpolation or extrapolation of learned targets. Considering the same target types (i.e. where only the angle *θ* is changed), targets will be located at approximately the intermediate positions between the trained ones. Regarding such cases, the policies can easily succeed in the reaching tasks by interpolating learned behaviours. By contrast, when the targets were changed to the frontal targets, most of the targets were no longer at the intermediate positions between the targets in the horizontal or sagittal targets. Therefore, policies have to extrapolate from learned behaviours, which is generally difficult. It can be observed that the arm was moved to the points where the target is projected onto the horizontal/sagittal plane. The policies attempted to reach the frontal and upper targets by interpolating the learned behaviours.

The endpoint trajectories show a characteristic feature as observed in human reaching movements. The trajectories were almost along straight lines to the targets. Although the trajectories were slightly drooping in the *z*-direction owing to gravity, the policy compensated most of the gravity effect. The gravity effect was not completely eliminated because of a trade-off with the reward term of energy minimization in (2.3). It has been reported that humans move their hand tips in a straight line to the target position [3]. This phenomenon has been reproduced computationally in previous studies [15,31]; nevertheless, the gravity force in these studies is compensated beforehand. By contrast, those policies reproduce the same phenomenon under the gravity that exists. This indicates that the learned movements reflect the skills of the policies, such as gravity compensation.

The fact that the policies failed in new targets (i.e. frontal and upper targets) is important for the purpose of this study. We aimed to demonstrate the generalization ability of the combined motor synergies for new targets; therefore, it is preferred that the new targets are exactly new for agents (policies or humans). The performance degradation in the policies in new targets implies that these targets were totally new for the policies. Therefore, if motor synergies generalize to these new targets by combination, we can conclude that the generalization ability emerged from the motor synergy combinations, not the policies. It is, in contrast, difficult to verify the same thing with human subjects who had already obtained motor synergies for various motor tasks. In addition, given recent developments in deep reinforcement learning, the same approach is expected to be applied to more complicated motor tasks.

### 4.2. Representation ability of motor synergies

Motor synergies are able to reconstruct the original actions (figure 3) and achieve the reaching task (figure 4). Only four motor synergies resulted in *R*^2^ > 0.95 in both the horizontal and sagittal targets. Moreover, the average target errors could be reduced to approximately 1 cm in both the horizontal and sagittal targets with only five motor synergies. It can also be observed that the trajectories of motor synergies (figure 4) resemble those of the policies (figure 2). Because a single movement consists of 100 actions of seven joints, the motor synergies significantly and efficiently reduce the dimensionality of the movements.

We observe the relationship between the reconstruction accuracy and performance. The selection of motor synergies with high reconstruction accuracy resulted in high performance, suggesting that the reconstruction accuracy could be used to determine the appropriate number of motor synergies for motor control. Previous studies that applied motor synergies to robot control employed reconstruction accuracy to determine the number of motor synergies to use [32–35]. However, the performance and *R*^2^-values did not completely correspond to each other. For example, the cases of three and four motor synergies showed almost double the difference in the reaching errors, whereas there were no such remarkable changes in *R*^2^-values, especially in the sagittal targets (figures 3*d* and 4*e*). A possible reason is the gap between signal and performance reconstructions as pointed out in [36].

Each repertory of the extracted motor synergies is considered to be specialized for the corresponding targets. Regarding the horizontal targets, the number of motor synergies from the horizontal policy remarkably affected the performance, whereas those from the sagittal policy hardly changed the performance (figure 4*b*). Considering the sagittal targets, the motor synergies from the sagittal policy affected the performance, whereas those from the horizontal policy did not (figure 4*e*). Moreover, neither was the repertory able to improve the performance in the frontal and upper targets by itself (figures 5 and 6). It is notable that the motor synergy repertoires from the horizontal targets themselves were not able to reach the upper targets, where only the height was different. Such a difference in the contribution of the motor synergies between the targets suggests that motor synergies are related to specific motor skills.

Focusing on each of the extracted motor synergies, we can observe that they encode task information efficiently. The waveforms of the motor synergies shown in figure 3*b*,*e* show the trend changes in *t*_target_ = 0.5 s. For example, in figure 3*b*, the joint torque four in synergy zero changes from negative to positive values at *t*_target_. Furthermore, considering figure 3*e*, joint torque two in synergy three rapidly decreases at *t*_target_. These trend changes show that the movement directions change at *t*_target_. Therefore, the motor synergies efficiently encoded the temporal information about the task, which were the changes in the reaching direction over time. This is in contrast to the other motor representations proposed in the robotics field, such as dynamic movement primitives (DMP) [19,20], which do not depend on specific tasks. Because the motor synergies encode specific skills of experts (e.g. humans and trained policies), they (the motor synergies) are considered suitable for handling a combination of multiple skills.

The motor synergies themselves and their activation patterns also seem to encode the task information. The amplitude of the motor synergy activities change in the cosine-like form for the angles of $q_{\mathrm{target}}$, according to figure 3*c*,*f*. Each motor synergy strongly activates at a particular target direction, and movements toward the intermediate directions between them can be represented by combinations of these motor synergies. Previous studies report similar activation patterns in human reaching movements [3,25,37] although the types of motor synergies are different. There are regular spatial patterns in motor synergy activities, which can improve the generalization ability of motor command generators by explicitly incorporating it into the design.

### 4.3. Generalization by combination of motor synergy repertories

It is demonstrated that the combination of multiple motor synergy repertories can be generalized to new targets, even in the new planes. Considering figures 5 and 6, the combination of four to five motor synergies from each of the horizontal and sagittal policies results in comparable performances to those of reinforcement learning (table 3). In addition, the trajectories are approximately straight lines, similar to those obtained by reinforcement learning (figures 2, 5 and 6). This suggests that the skills of the original policies, such as gravity compensation, are also reproduced. The same is suggested by the fact that a straight trajectory started to appear early in the optimization process (figures 5*d* and 6*d*), contrary to reinforcement learning, where the complex curved trajectories are generated at the beginning of motor learning (figure 2*c*,*g*).

The performance was remarkably improved only when both repertories of the motor synergies from the horizontal/sagittal policies are combined (figures 5 and 6). Considering the frontal targets, when only single repertories were used (i.e. *L*_*h*_ = 0 or *L*_*s*_ = 0) the minimum error was 6.49 cm at *L*_*h*_ = 5. The trajectories do not seem to have reached the targets successfully in most cases (figure 5*e*,*f*). Considering the case where *L*_*h*_ = *L*_*s*_ = 3, the error was reduced to 4.24 cm although the number of motor synergies were approximately the same as in the above case. The error was further reduced by adding one motor synergy from each of the two repertories, whereas it hardly changed when two motor synergies were added from single repertories. A similar tendency was also observed in the upper targets. Regarding these results, the essential factor for generalization is not to simply increase the motor synergies; nonetheless, it is to combine multiple repertories of the motor synergies.

Motor synergies, however, were not always effective for different arm models. The motor synergies extracted from the longer-arm model were only able to show lower performance than that of the original arm model (figures 5*a* and 7*d*). This would be because the motor synergies depend on the inverse kinematics of the arm, which varies for different arm models. By contrast, the motor synergies from the shorter-arm model resulted in a similar performance to the original arm model. Thus, motor synergies may be transferable for different bodies in some cases and enable the new bodies to perform the same task. Whether a repertory of motor synergies is transferable for another body is not simple and remains as future work. Besides, in both cases, we can yet observe a similar tendency to the previous subsection that the performance was only improved when both repertories of the motor synergies were combined.

Considering the combination of multiple motor synergy repertories, linearity plays an important role. Linearity makes it possible to extend the current repertory of motor synergies by adding other motor synergies. This study directly demonstrates this using the optimization of the frontal and upper targets (figures 5*c* and 6*c*). The motor synergy repertories extracted from the horizontal and sagittal policies enable *x*–*y* and *x*–*z* directional reaching, respectively. Each repertory itself cannot realize *y*–*z* directional reaching movements; however, combining them, a new skill that can perform *y*–*z* directional reaching can be realized. Whereas there exists a study that addresses the extension of motor synergy repertories [38], our study extends the repertories with motor synergies that are extracted from individually learned movements.

These findings can also contribute to machine learning and robotics. Our results suggest the possibility that a robot can construct new movements by re-using previously acquired skills through motor synergies. Even if motor synergies may not be able to work in other robots that have totally different kinematics, that fact can be beneficial toward the scalability of intelligent robots. Therefore, it will improve the efficiency of motor learning, although there is a labour of motor synergy extraction.

### 4.4. Comparison with the findings in neuroscience

Although it is not our goal to describe the actual adaptation process in humans, it is possible to compare our results with past findings in neuroscience. Previous studies have suggested that humans employ the superposition of motor synergies to generate new movements. Ivanenko *et al.* and Clark *et al.* reported that approximately five motor synergies were able to represent human locomotion [39,40]. Moreover, Ivanenko *et al.* reported that additional motor synergies make it possible to represent other types of movements such as straddling-obstacle movements [41]. Similar findings are reported in multi-directional reaching tasks [4]. Although our study did not consider the timing of motor synergy activation, as in the above studies, we computationally reproduced a similar phenomenon in which new movements were generated by extending motor synergy repertoires.

Our findings are also closely related to the shared synergies and motor synergies commonly observed in movements for different tasks [6,9]. Scano *et al.* showed that similar motor synergy structures were observed between reaching tasks with various target positions [9]. Israely *et al.* demonstrated that a set of motor synergies in a reaching task can account for other directional movements [8]. Those studies have shown the existence of the shared synergies; by contrast, our study demonstrates a process in which motor synergies are re-used for new targets to function as shared synergies. In this study, the motor synergies are not intended to be shared with different types of targets. This is in contrast to other studies that directly designed shared-synergy structures [21,42].

However, there are differences between our method and the human adaptation process. One difference is that we only treated joint-level synergies, whereas human studies usually consider muscle-level synergies. As the numbers of muscles are usually more than those of joints, higher dimensionality needs to be treated than that of ours in muscle-level synergies. Muscle-level synergies also consider redundancy of antagonistic muscles, which do not appear in joint-level synergies. On the other hand, the tasks in this study also have redundancy and nonlinearity due to the inverse kinematics. Moreover, previous studies pointed out that joint-level synergies are highly correlated with muscle-level synergies [43,44]. These suggest that our results can also be applied to muscle-level synergies of more complex musculoskeletal systems.

Another difference is that in humans, the motor synergies themselves can change while learning new tasks. Motor synergy structures have been reported to gradually change during development or motor skill learning [45–47]. It has also been reported that the number of motor synergies increases in experts who have experienced long-term motor training in specific tasks [48]. By contrast, our method does not have a function that makes it possible to modify the motor synergies after extraction. Although the modification of motor synergies is slow and small [11,49], fine-tuning the motor synergy structure may improve the final performance in more complex tasks. In addition, we used spatiotemporal motor synergies, in which motor activities were feed-forwardly determined, and sensory feedback played an important role in the motor pattern generation in the central nervous system [14,50–53]. Although this study addresses a situation where feed-forward motor commands are sufficient, the extension to sensorimotor feedback remains as future study.

It should be noted that the reaching task in this study is complex even compared with related works that used musculoskeletal systems because we treated a three-dimensional task. Although recent studies have dealt with simulated musculoskeletal systems with many muscles [22,54,55], most studies only handled planar physics, in which the rotational directions of joints are always constant (i.e. perpendicular to the plane). By contrast, in three-dimensional reaching, the rotational axes of joints vary depending on other joint angles; for example, the rotation direction of the elbow joint changes depending on shoulder-joint angles. Owing to such difficulty, there are still only a few studies that dealt with three-dimensional tasks with motor synergies [56].

## 5. Conclusion

This study reveals that a linear combination of motor synergies extracted from multiple policies can be generalized to new targets that cannot be achieved with either the original policies or single motor synergy repertories. We have demonstrated a new framework that consists of (i) motor learning to acquire individual skills, (ii) motor synergy extraction from the skills, and (iii) generalization to new targets by combining the motor synergies and optimizing their activities. We also found that the motor synergies efficiently encoded the movements achieving the tasks, and the representation ability of the motor synergies could be easily extended by superposition owing to the linearity. New targets in new planes could be realized with errors within 1 cm, even for the new plane that was perpendicular to the learned planes. These computational results suggest the advantages of motor synergies for the generalization of motor skills.

We believe this is the first study that has succeeded in motor synergy generalization for new targets in multi-directional reaching with a full seven-d.f. arm under gravity, a realistic mechanical environment for the motor task. It was previously difficult to be verified with the motion analysis performed by human subjects who had already obtained motor synergies for various motor tasks. Moreover, our results provide important contributions to machine learning and robotics field. Our finding highlights that the generalization ability of motor synergies supports the employment of motor synergy representations in the computational learning process.

## Data Availability

The codes to run simulations are available at https://doi.org/10.5281/zenodo.6395483.
